# Implementation and Analysis of a Wireless Sensor Network-Based Pet Location Monitoring System for Domestic Scenarios

**DOI:** 10.3390/s16091384

**Published:** 2016-08-30

**Authors:** Erik Aguirre, Peio Lopez-Iturri, Leyre Azpilicueta, José Javier Astrain, Jesús Villadangos, Daniel Santesteban, Francisco Falcone

**Affiliations:** 1 Electrical and Electronic Engineering Department, Public University of Navarre, 31006 Pamplona, Spain; aguirrerik@gmail.com (E.A.); peio.lopez@unavarra.es (P.L.-I.); santesteban.58544@e.unavarra.es (D.S.); 2 School of Engineering and Sciences, Tecnologico de Monterrey, 64849 Monterrey, Mexico; leyre.azpilicueta@itesm.mx; 3 Mathematical Engineering and Computer Science Department, Institute of Smart Cities, Public University of Navarre, 31006 Pamplona, Spain; josej.astrain@unavarra.es (J.J.A.); jesusv@unavarra.es (J.V.)

**Keywords:** dog monitoring, WSN, IoT, 3D ray launching, ZigBee

## Abstract

The flexibility of new age wireless networks and the variety of sensors to measure a high number of variables, lead to new scenarios where anything can be monitored by small electronic devices, thereby implementing Wireless Sensor Networks (WSN). Thanks to ZigBee, RFID or WiFi networks the precise location of humans or animals as well as some biological parameters can be known in real-time. However, since wireless sensors must be attached to biological tissues and they are highly dispersive, propagation of electromagnetic waves must be studied to deploy an efficient and well-working network. The main goal of this work is to study the influence of wireless channel limitations in the operation of a specific pet monitoring system, validated at physical channel as well as at functional level. In this sense, radio wave propagation produced by ZigBee devices operating at the ISM 2.4 GHz band is studied through an in-house developed 3D Ray Launching simulation tool, in order to analyze coverage/capacity relations for the optimal system selection as well as deployment strategy in terms of number of transceivers and location. Furthermore, a simplified dog model is developed for simulation code, considering not only its morphology but also its dielectric properties. Relevant wireless channel information such as power distribution, power delay profile and delay spread graphs are obtained providing an extensive wireless channel analysis. A functional dog monitoring system is presented, operating over the implemented ZigBee network and providing real time information to Android based devices. The proposed system can be scaled in order to consider different types of domestic pets as well as new user based functionalities.

## 1. Introduction

Nowadays, monitoring of objects as well as of living beings can be easily carried out thanks to a wide variety of transceivers working together with increasingly compact devices and the use of different wireless communication standards. These systems fall within the scope of the Internet of Things (IoT), where devices are connected to the internet and the information is sent without the interaction of human beings. Thus, a high variety of variables can be monitored in real time and in the case of living beings, their physiological parameters or location can be accurately measured.

In this sense, a high variety of person-oriented systems are being developed within the framework of e-health systems and therefore, most of these technologies are being extrapolated to farming and pet wellness applications. Monitoring of physiological parameters as ECG [1], pulse [2] or blood pressure [3], for patients, elderly people or athletes as well as their location [4], are the most common data obtained in this kind of systems.

In relation to animal tracking and wellness, numerous identification systems have been developed over the years, specially for wildlife tracking and analysis, attaching radio transmitters to animals to monitor their location, behavior or migratory habits [5,6]. For farm animals or pets some identification systems have also been developed and deployed, although these systems are not based on wireless networks and therefore owners must extract the information indirectly.

Nevertheless, novel farm animal monitoring systems based on WSN have been studied in recent years, in [7] a WSN is proposed for animal monitoring, in [8] the location of cows is monitored thanks to GPS location system and GSM telephony and in [9] a ZigBee-Based health monitoring system is presented, which takes data through rumination, heart rate, temperature and humidity sensors. Going one step further, in [10] a Livestock Monitoring System (LMS) is presented, where thanks to an integrated system for animal monitoring of their environment, production, growth and health, the production efficiency in farming is improved.

When WSN are developed for pet-oriented applications, the main purpose of these systems is animal wellness and security. Thus, veterinary systems where photophethysmogram (PPG) and electrocardiogram (ECG) information are recorded wirelessly are presented in [11,12], a remote feeding system is developed in [13] and a system capable to recognize dog behavior thanks to an accelerometer attached to the canine is shown in [14]. Dogs also play a useful role in rescue operations and therefore some devices are attached to a canine in [15] to obtain not only useful information about dog health (ECG, PPG), but also environmental information about location or the presence of gas. Table 1 presents a comparison of different wireless animal monitoring systems. It is worth noting that no specific application or system has been identified for pet location and monitoring applications within domestic indoor scenarios. The proposed system therefore provides a pet location and monitoring system, which provides an interactive context in order to retrieve parameters such as pet location, biomedical signal retrieval or behavior patterns, among others.

Notwithstanding, these kind of systems must be deployed after an extensive radio-planning study to implement a robust and efficient Wireless Sensor Network (WSN), especially when the number of animals is high, they are in wide areas or they are inside complex places from the electromagnetic point of view.

In this work an indoor canine location and monitoring system for home environments is presented. An in-house 3D Ray Launching method is used to study indoor wireless channel performance inside a home when the Zigbee WSN is deployed and a receiver is attached to a dog. The main goal is the analysis of the influence of wireless propagation limitations in the implementation of a specific pet monitoring system, which has been implemented and tested. For this purpose, a simplified dog model has been developed which is compatible with the simulation tool accounting for its morphology and dielectric properties, considering that the wireless transceiver is inevitably attached to biological tissues and they are highly absorptive and dispersive. Radio planning analysis is performed, based on deterministic 3D Ray Launching code in order to analyze coverage/capacity relations, aiding in system election as well as on the number of required transceivers and their potential location within the scenario under analysis. Validation measurements have been carried out by attaching a Xbee device to a real dog to calibrate the simulation tool and compare obtained data with theoretical results. An android-based location application operating within the scenario is presented and tested in order to provide an interactive indoor pet location and monitoring system.

## 2. Simulation Scenario and Simplified Dog Model

As previously mentioned, an in-house developed 3D Ray Launching (3D RL) code has been employed in order to perform radioplanning analysis and coverage/capacity estimations in order to provide system wireless connectivity, previously tested in a wide range of application [16]. Moreover, the biological tissues can be considered, by means of a specific simplified human body model implemented for use within the 3D RL code [17,18].

The 3D RL technique has been selected provided an adequate trade-off between accuracy and computational cost. Simulation techniques can be divided in two main groups, empirical and deterministic methods. Empirical methods [19,20] are site-specifi, measurement based and therefore, their results are strongly related to the framework scenario and original conditions. Thereofre, such techniques are time efficient but inaccurate when compared to deterministic techniques as FDTD, MOM, Ray Launching or Ray Tracing [21,22]. On the other hand, deterministic techniques are based on numerical approaches to the resolution of Maxwell’s equations and therefore, their computational complexity is high. However, Geometrical Optics based techniques (Ray tracing) show a good balance between both: computation time and accuracy.

The algorithm has been implemented in Matlab and is based on Geometrical Optics (GO) and Geometrical Theory of Diffraction (GTD). In order to enhance GO theory, the uniform extension of the GTD (UTD) is used with the diffracted rays, which are introduced to remove field discontinuities and to give proper field corrections, especially in the zero-field regions predicted by GO.

The input parameters in the algorithm are the angular and spatial resolution, which determine the accuracy of the model. A bundle of rays are considered in a finite sample of the possible directions of the propagation from the transmitter, and a ray is launched for each direction. When a ray impacts on an object, a reflecting ray is generated, and when a ray impacts an edge, a new family of diffracted rays is generated. Rays are launched at an elevation angle θ and with an azimuth angle Φ, as defined in the usual Cartesian coordinate system.

It is worth noting that a grid is defined in the simulation space, and all the parameters of the propagating rays are stored in each cuboid in the space. After that, with this stored parameters, simulation results such as received power or power delay profiles for each spatial point in the three dimensional scenario can be obtained.

Antenna patterns are taken into account in the algorithm to include the effects of antenna beamwidth in both azimuth and elevation. The material properties for all the elements within the scenario are also taken into account, given the dielectric constant and permittivity at the frequency range of operation of the system under analysis.

**Figure 1:**
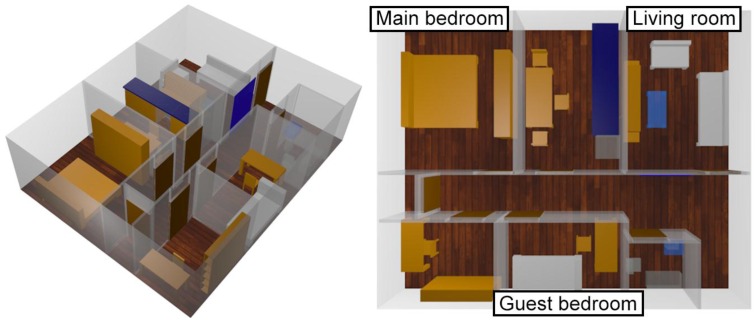
3D view of the simulated measured scenario.

In this case a one story home (Figure 1) has been simulated, including furniture (chairs, tables, mirrors, beds, etc.) and taking under consideration not only the morphology of the scenario but also the dielectric properties of all the objects inside it. The scenario surface is 65 m^2^, divided in eight rooms. Three antennas have been placed within the scenario: one in the ceiling of living room, one in the main bedroom and one in the guest bedroom. In addition, another transceiver is placed simplified dog model. This configuration is chosen since the indoor canine tracking system requires at least three transmitters to carry out the triangulation process. Moreover, typical wireless motes can provide coverage levels, by means of experimental validation, in the order to 20–40 m^2^, as a function of object density. In this way, coverage conditions can be fulfilled, whilst enabling location capabilities.

Table 1 shows the employed simulation parameter, following conventional Xbee mote specifications (802.15, ZigBee device), the ones which will be used in measurement validations. ZigBee has been chosen as the base of the presented system considering its low energy consumption characteristics. The device attached to the dog must be as small as possible to provide high comfort levels and therefore the system must be energetically efficient to allow the use of small batteries. On the other hand, the ZigBee data rate is lower than, for example, 802.11 system based devices, which is not relevant in the proposed system, since the required transmitted information volume is potentially low. It is also worth noting that the final system design, after prototyping stage, can be more ergonomic, by including chip or conformal antennas if required.

**Figure 2:**
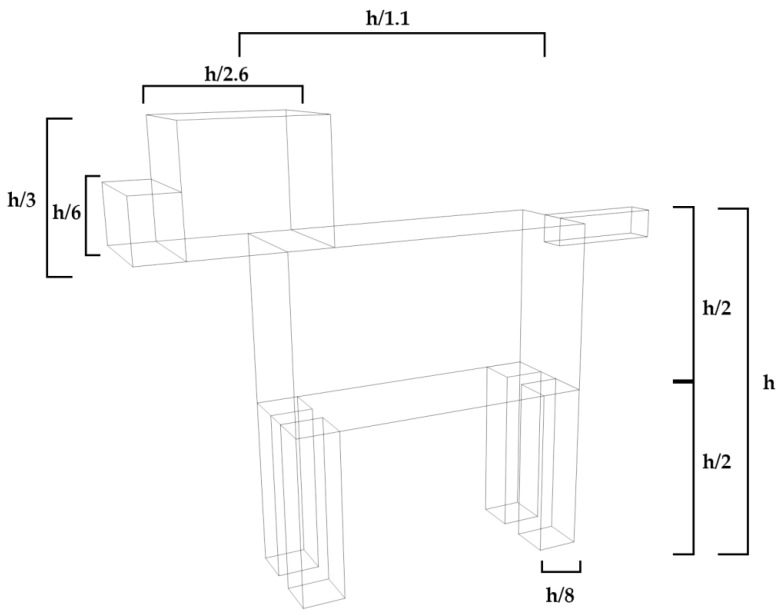
Proportions of simplified dog model according with chosen height for medium size dog.

The implemented dog model is depicted in Figure 2. This model is compatible with the 3D RL simulation technique and includes the dielectric characteristics and proportions of a real dog, which have been extrapolated from measurement results related with cattle as well as characterization of human bodies, with dielectric constant values of the outer skin layer (in principle the most relevant) in order of ε_r_ ≈ 4 for the frequency of operation of the employed wireless communication system. Maintaining these proportions is not a trivial task, considering that every breed of dog has its own characteristics. However, most dogs can be classified in five different groups and therefore, the developed simplified canine model has been programmed to allow the generation of dogs of different proportions, from mini-dogs like Chihuahuas to giant-dogs like St. Bernards.

Once the kind of dog has been chosen in the simulation code, its height is defined and the dimensions of all elements of the dog are automatically calculated. In Figure 2 proportions and its relation with the height of the dog are depicted for a medium size canine breed.

**Figure 3:**
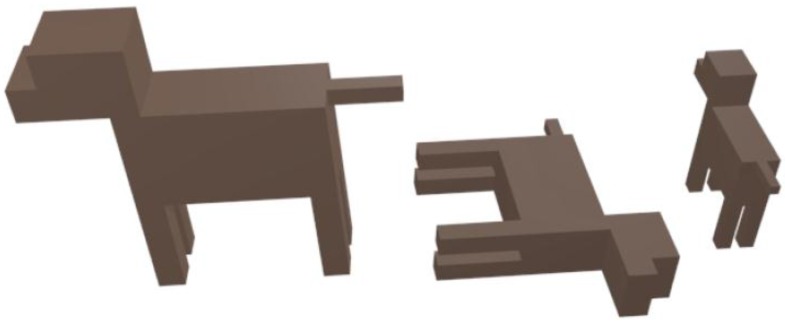
Example of the simplified dog model in different position and with different sizes.

The implemented simplified dog model is programmed to allow the consideration of not only different sizes but also different positions to be as adaptable as possible, considering the potential impact in propagation losses. As an example, in Figure 3 the dog model is depicted in different positions which can be considered within the simulation code.

Since the communication system employed is a ZigBee Wireless Personal Area Network (WPAN), transceiver simulation parameters match with real device characteristics, corresponding to Xbee devices which have been used for validation measurements. In Table 2 these parameters as well as resolution values used in simulation runs are shown. In any case, transceiver selection is in principle flexible, allowing if required other solutions based on 802.15 systems or even combined with 802.11 systems. The election of ZigBee in this case is based initially on the flexibility to implement low-cost and largely scalable networks, which can be easily connected via gateway to an external application server. The solution however can be adapted to other communication systems or even into a multi-system solution (combining for example ZigBee with WLAN connectivity to provide interaction between mobile devices and motes within the wireless sensor networks), depending on the functionalities to be developed in the future.

**Figure 4:**
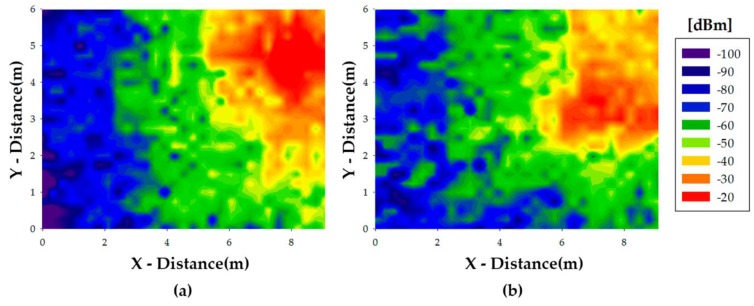
Power distribution in the scenario when transmitter is placed in the living room for an empty living room (**a**) and when the dog is inside the lounge (**b**).

In Figure 4 power distribution results for the home floor when the living room is empty and when a dog is within the living room are presented. In this case the antenna has been placed in a lower height, over the glass table which is placed in the center of the living room. In both cases, when the transceiver is attached to the dog and when it is over the table the height is 0.3 m, although simulation results have been obtained for the complete scenario volume. Since the transmitter is placed in this room, most of the energy is confined in this area. However, power levels obtained in the rest of the scenario are compatible with usual Xbee receiver sensitivity thresholds in the range of −100 dBm. The difference between both situations is also noticeable, since more power is distributed when the room is empty and it is not absorbed by the dog. In any case, the difference between both images is more visible given that the transmitter is inside the same room as the dog and the presented power planes are at the dog height. As it can be seen from the estimation of received power levels within the scenario, an optimal configuration would require 2 transceivers within the scenario to satisfy coverage conditions for the domestic pet-static infrastructure links, implying an approximate node density of (1 node/30–40 m^2^). These values are dependent on the specific configuration of the scenario, although the node density, for the case of large complexity (i.e., relevant number of walls and furniture elements), provides a radioplanning estimate to perform initial network deployment. This node density is one of the relevant outcomes of the radioplanning process, providing an initial network deployment estimation, which requires fine tuning as a function of site-specific details.

**Figure 5:**
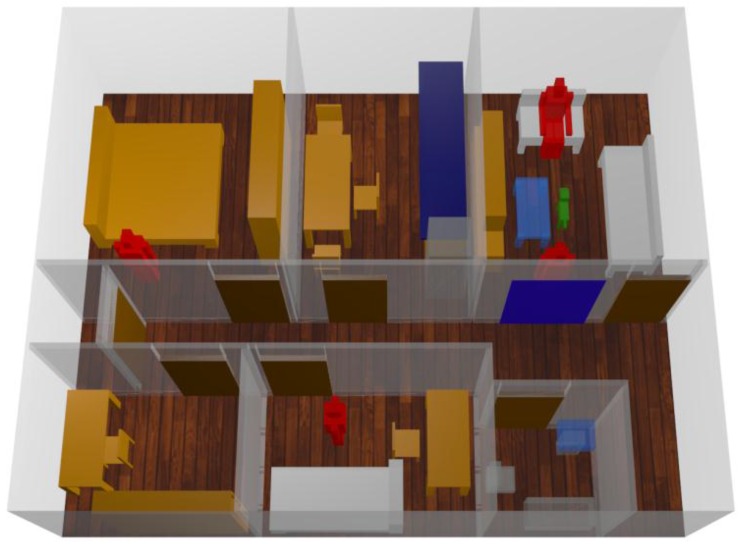
Simulated scenario when four human bodies models are introduced in different locations.

**Figure 6:**
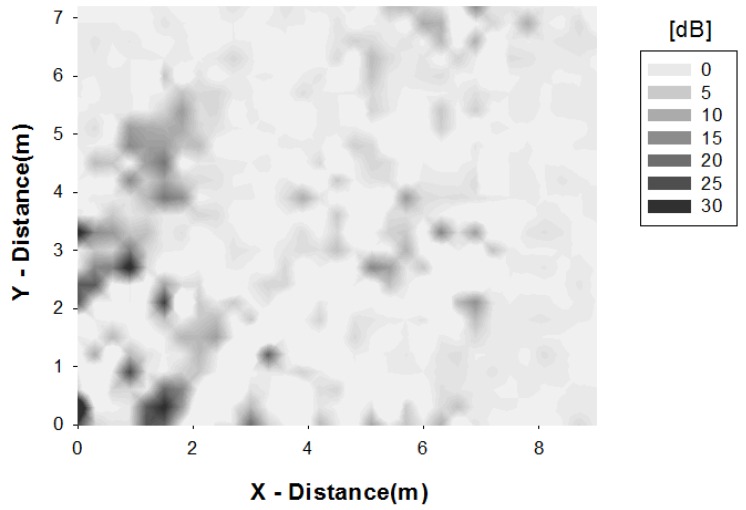
Comparison of receiver power when human body models are introduced in the home.

In order to consider a more general case, the scenario has also been simulated introducing four human body models inside the home (Figure 5). Two of them are placed in the living room next to the dog model and other two are distributed in different stances of the scenario. In Figure 6 the difference between simulation results when only the dog is inside the home and when humans are introduced is depicted. The largest difference is shown in the furthest room of the home, reaching 30 dB values. This behavior is the consequence of introducing human bodies between transmitter area and this room, due to the fact that human bodies have absorbed and reflected the energy partially.

In any case the influence of human bodies is low considering that the transmitter is not attached to them and that the number of people which usually can be found inside this kind of scenario is not large enough. Therefore, if a human monitoring system or an area of high human presence density were considered, the study of the presence of human bodies would be essential to determine the adequate performance of the communication system.

When a wireless sensor network is deployed the accuracy of data transmission must be assured to achieve the best possible user experience, provided by bit erro rate (BER) estimations. For the case of Quaternary Phase Shift Keying (QPSK) modulation (a typical modulation scheme employed in ZigBee as well as in 802.15 and 8021.11 systems), the approximate value of bit error probability is given by:

(1) $$P_{b}=Q\left(\sqrt{\frac{2E_{b}}{N_{0}}}\right)$$

where P_b_ is the bit error probability, N_0_ is the overall interference power spectral density (given by internal interference, external interference and thermal noise) in the scenario and E_b_ is the energy per bit, given by:

(2) $$E_{b}=\frac{P_{\text{Rx}}}{R_{b}}$$

where P_Rx_ is the received power and R_b_ is the transmission bit rate. Hence, BER strongly depends on the transmission speed, noise level in the scenario and received power.

**Figure 7:**
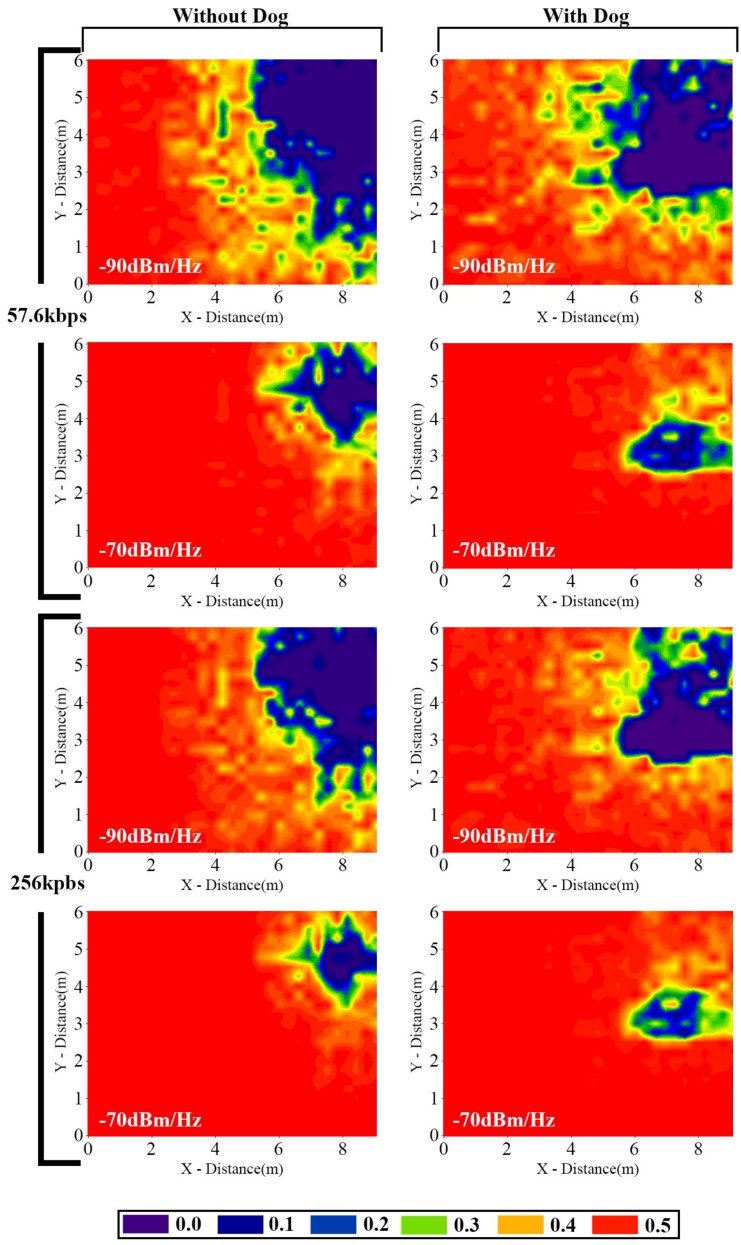
BER results considering different bit rates and noise levels for the antenna placed in the living room.

In Figure 7 BER results are depicted for different conditions when the transmitter is placed in the living room. Not only noise levels have been modified, but also bit rates and the presence of the dog have been taken under consideration. As expected, in any condition when noise level or transmission rate is reduced, low BER area is larger and therefore, longer range communication links can be established. Since one of the variables which determines BER is the received transmission power, energy lost by obstacles and multipath behavior of electromagnetic wave imply chenges in BER values all over the scenario. The presence of the dog decreases the overall power in the living room and low BER area is reduced when it is considered. The results depicted consider very high interference levels, which are not the usual case within a domestic environment. The usual practical case exhibits noise power spectral densities in the order of −120 dBm/Hz to −100 dBm/Hz. In this case, the main condition of operation is provided by coverage thresholds, which are a function of the requested bit rate and the sensititivy threshold. Taking into account the results for coverage level depicted in Figure 4, each one of the employed ZigBee transceivers provides coverage levels in an approximate area of 40 m^2^, as a function of the elements within the scenario. Therefore, in the domestic scenario under analysis, a network deployment of 1–3 transceivers per floor will satisfy initial coverage needs in order to enable bi-directional real time communication between the static transceivers and the wearable device located on the pet. Therefore, not only received power level thresholds are satisfied, but also requirements in terms of BER, leading to coverage/capacity relations that hold for the scenario under analysis, with a node density of (1 node/30–40 m^2^) in order to satisfy both conditions for Xbee motes operating al 0 dBm transmission power levels. As in the previously derived node density values, these results provide relevant information related with the node radioplanning estimations.

## 3. Experimental Results and Implemented Application

Once the 3D Ray Launching simulation technique has been introduced, experimental validation results as well as the implementation of an dog indoor tracking application is presented. The goal is to validate the proposed simulation model of the pet (in this case, a parametrizable dog model), prior to testing the proposed monitoring application.

**Figure 8:**
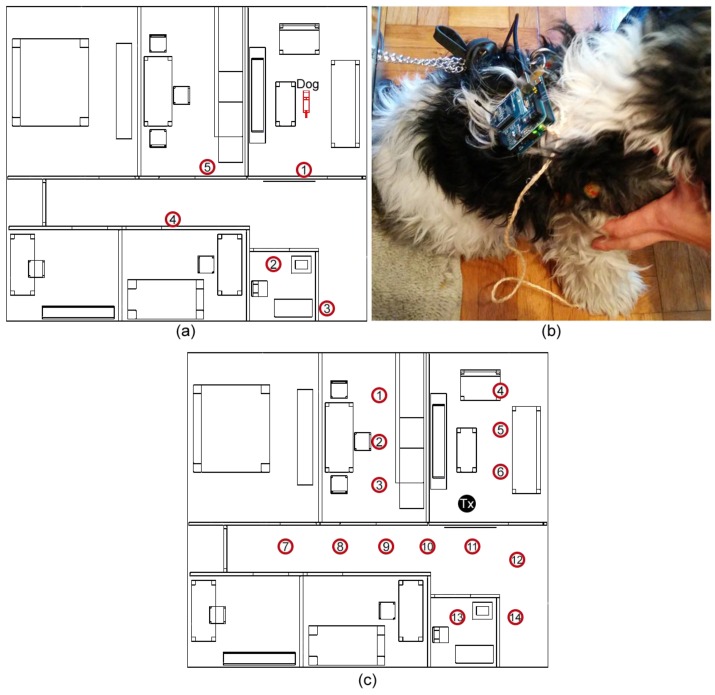
(**a**) Measurement points distributed around the home and the location of the dog; (**b**) Xbee device attached to the dog for measurements; (**c**) Measurement points distributed around the home when the dog is not considered.

Experimental results are obtained by attaching to a dog a Xbee device working over an implemented Arduino software platform (Figure 8b). In this case dog has been placed in the living room and simulation and measurement results have been obtained all over the home under analysis. Measurement results have been obtained with the aid of an Agilent FieldFox N9912A portable spectrum analyzer. In order to validate the results obtained from the deterministic 3D RL code, measurements have been performed with the aid of a portable spectrum analyzer, which is necessary due to the fact that measurement error is very small (in the order of 0.1 dB) with adequate bandwidth settings.

Received power levels can also be obtained (by means of Received Signal Strength Indication signals, RSSI) with the internal circuits of the ZigBee mote transceiver, but in this case, measurement errors are relatively high, within the order of 5 dB–8 dB. Therefore, wireless planning requires calibration in terms of precise spectral measurements and real operation can be estimated by considering a constant degradation off-set corresponding the RSSI detection circuits [23]. Five measurement points are defined in different rooms of the home, which are depicted in Figure 8a. In addition, 14 measurements have been carried out placing the antenna in the living room, without the presence of the dog (Figure 8c).

**Figure 9:**
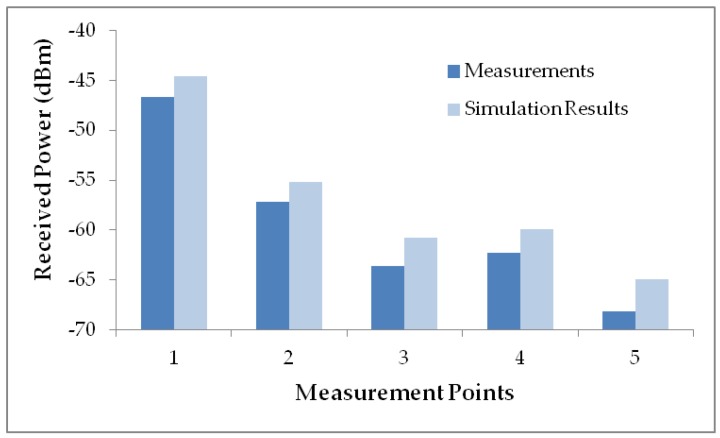
Measurement vs simulation results when the Xbee prototype is attached to the dog within the scenario under analysis.

In Figure 9 both, measurement and simulation results are compared for the first case, concluding that the simulation tool coupled with the simplified canine model, provides accurate results with a maximum error of 5 dB, a mean error of 2.52 dB and a standard deviation of 0.48 dB. In any case, it can be seen that the derived power value is far away from the sensitivity threshold and therefore, good performance of the ZigBee system can be expected, with the initial consideration of 1–3 static transceivers per household floor, as a function of the specific propagation losses of the considered scenario.

**Figure 10:**
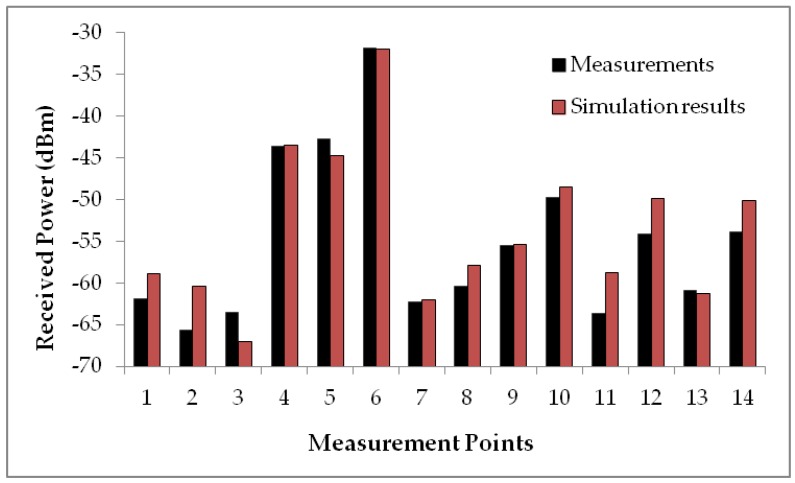
Measurement vs simulation results when the dog is not located inside the room.

In Figure 10 measurement data compared with simulation results, when the transmitter is not attached to the dog, are depicted and in this case, the transmitter has been placed at a height of 0.8 m High accuracy is observed, with error values of 1.37 dB and a standard deviation of 2.55 dB. When two measurement results are compared, the influence of the dog is noticeable considering that received power values are in general higher within the considered stances when the home is empty. The influence of the furniture is also observable if the stances next to the living room are studied. As it can be seen in Figure 10, the received power level of point 11 is lower than the two points placed next to it (10 and 12) as a consequence of the power reflected by the mirror located between transmitter and the measurement point. For point 5 in Figure 9 and points 1, 2 and 3 in Figure 10, received power is lower in comparison with further points since most of the energy is reflected or absorbed by the furniture disposed in the middle wall of both stances.

**Figure 11:**
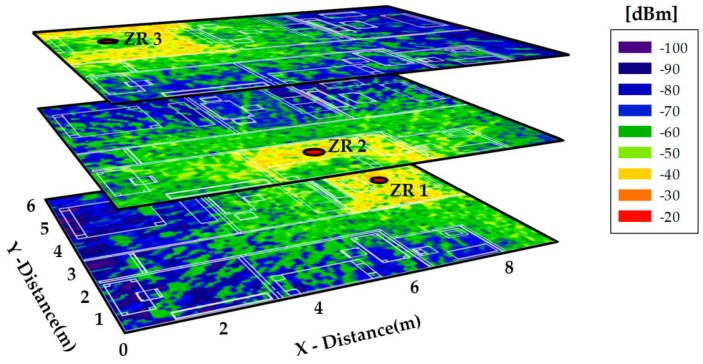
Power distribution in the scenario when transmitters are placed in the ceiling of living room (ZR1), guess bedroom (ZR2) and main bedroom (ZR3).

As aforementioned, the dog location system works triangulating the received power of three transmitter or Zigbee Routers (ZB) distributed in different rooms of the home. Therefore, three different simulations have been launched considering those antennas to show the influence of furniture and the household topology. Simulation results for these configurations are depicted in Figure 11.

These results can be used to calibrate the location system since power distribution values within the complete scenario are obtained. Besides, the high influence of home distribution and furniture is noticeable. As an example, in main and guest rooms the influence of bed is evident considering that when the situation of the bed is analyzed, a decrease in received power level is shown. Finally, it can be concluded that in the home the system will have enough power to carry out the triangulation process, considering that power in the complete area is higher than −100 dBm, above the typical receiver sensitivity value for ZigBee systems.

**Figure 12:**
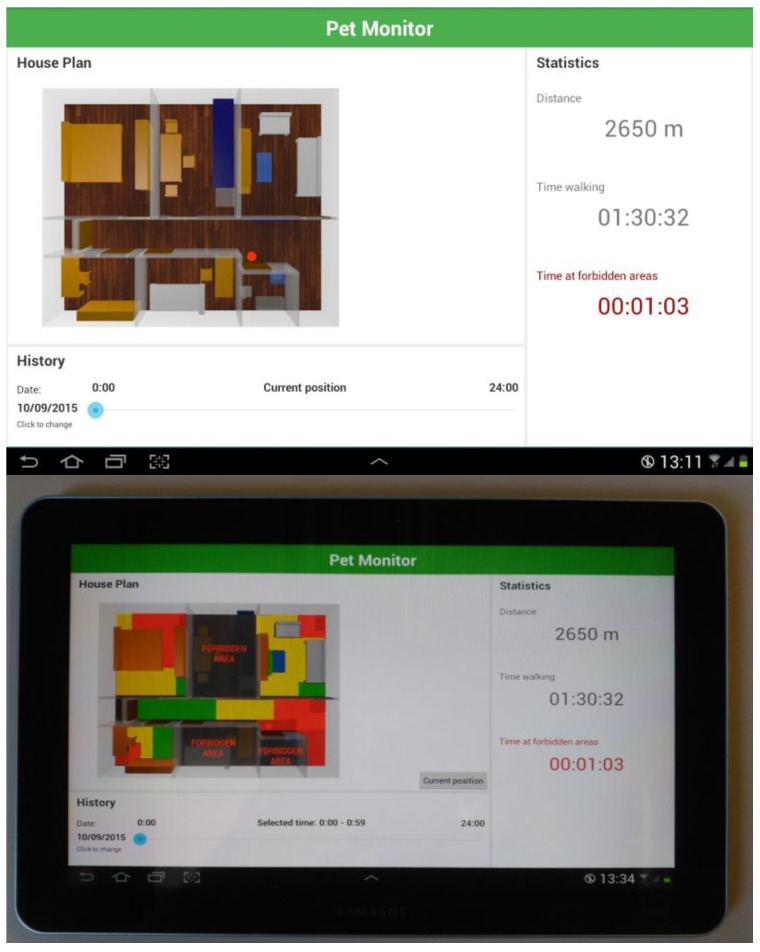
Implemented android based application for the dog monitoring inside a home.

In order to provide pet monitoring functionalities, an in-house dog monitoring system is developed for android devices based on the deployed ZigBee network. A screenshot of implemented application is shown in Figure 12, where the distance walked by the dog can be easily known and the place where it is as well as the time that it has been in banned places can be monitored. The dog monitoring system consists on a set of wireless devices and a software component. The hardware includes a ZigBee device in charge of data communication and RSS (received signal strength) monitoring, a ZigBee sink in charge of data collection and command execution, an Android-based tablet in charge of data presentation, and a mini-PC, which includes the information system.

The dog carries in its harness the Arduino device, which includes a ZigBee communication module. The monitoring of the Received Signal Strength Indicator (RSSI) allows to determine the location of the dog. This value is obtained by the ZigBee transceiver each time a message is received. The message also includes the identifier of the ZigBee node. This information (ID + RSSI) is sent periodically or on demand to the sink node in order to provide the dog location. The Arduino device allows the operation on real-time connected mode or even, on unconnected mode. The first mode is used for real-time data transmission to the sink node, while the second-one is used for off-line dog tracking. The energy consumption is higher for the first mode, so an appropriate and limited use of this operation mode is recommended. The Arduino device includes a ZigBee communication module to minimize the energy consumption, since its consumption is lower than this of WiFi. Figure 8b illustrates the devices used.

**Figure 13:**
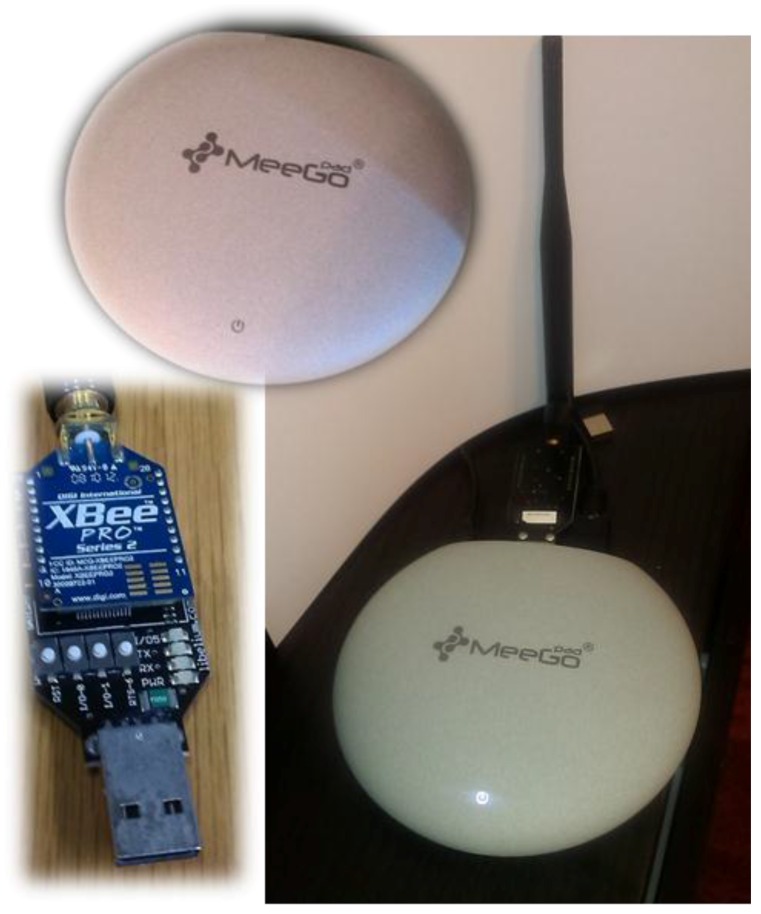
Infrastructure’s hardware, including XBee Pro ZigBee Motes and MeeGo Mini PC sink device.

**Figure 14:**
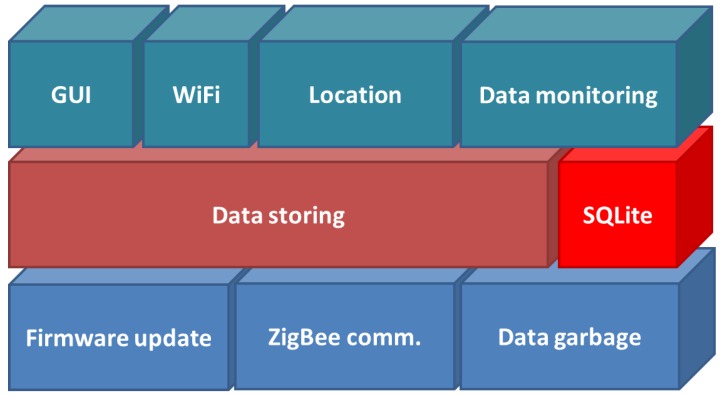
Middleware architecture.

The infrastructure includes the mini-PC and the ZigBee sink. In this case, we have chosen a MeeGoPad T04 Windows 10 based mini-PC. It includes a quadcore Intel X5-Z8300 Cherry Trail processor and 2 GB of RAM memory, WiFi b/g/n and two USB ports. We connect a ZigBee sink to its USB 2.0 port and then we can interact with the Arduino device located at the dog’s harness. We have developed a small and simple protocol to interact with the Arduino device. This protocol includes functionalities as the selection of the operation modes: location, tracking, rest; the battery level monitoring of the device; the firmware upload; the device diagnosis; and even more. The mini-PC runs a small relational database management system called SQLite (www.sqlite.org), which is embedded into the software. The C++−based software developed is a middleware in charge of data location storing into the database, data presentation on the graphical user interface GUI, and device management. Figure 13 shows the hardware, while Figure 14 shows the software architecture.

The middleware (see Figure 14) includes at its lower layer the basic functionalities needed to communicate the Arduino device with the mini-PC (ZigBee communication), collect the sensed data (Data garbage) and the remote update of the firmware when needed (firmware update). The middle layer provides data storing into a relational database. In this case SQLite, that is absolutely embedded with the code in charge of data storing. The upper layer of the middleware provides the graphic user interface (GUI) functionalities. The WiFi communication module allows the communication with the Android device (tablet), allowing the interaction with the GUI module. Finally, the location and tracking of the pet is performed by the Location and Data monitoring modules. The location module applies a set of fuzzy rules to the data collected by the Arduino device, and stored into the database, and estimates the location of the dog. Data monitoring includes the tracking of the dog, and also the statistical analysis of its behavior.

Data presentation can be performed over the TV screen (the mini-PC has an HDMI connector) and a wireless keyboard/pointer device, or over an android-based tablet via WiFi communication. The GUI has been developed on HTML 5, since that allows its use on different devices. Given the simplicity of the GUI, and the low complexity of the functionalities required, HTML is a good choice for this app.

**Figure 15:**
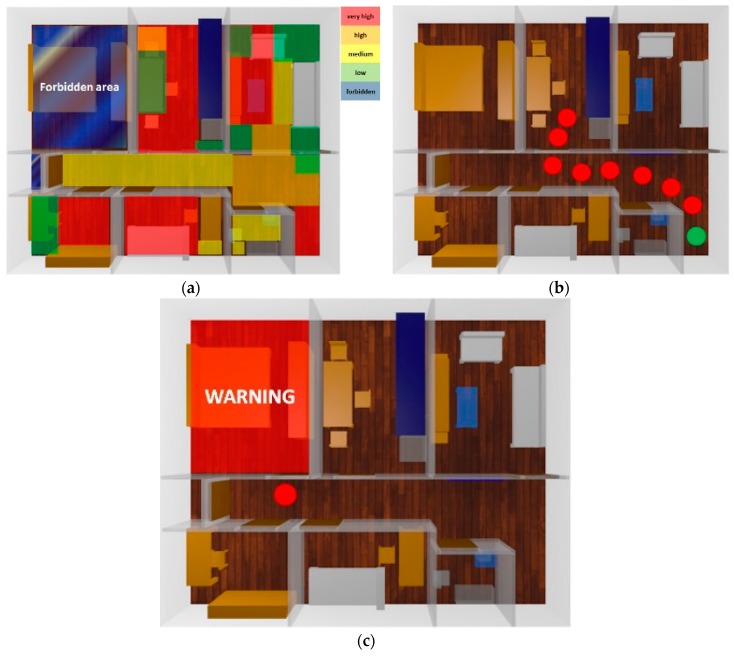
App functionalities: heat map (**a**), dog tracking (**b**) and forbidden area intrusion (**c**).

Figure 15 shows a heat map, which displays the location of the dog along the time period selected by the user. The user can monitor the movement of the dog anywhere from the past week to the past hour, the distance traveled, as well as the specific time and number of times the dog has entered a forbidden zone.

The location estimation is performed thanks to the ZigBee coverage. The ZigBee module of the Arduino device (mote) placed at the harness allows the measure of the RSSI values and then estimate the localization of the dog. Fuzzy location using RSSI values is a rough but reliable method that can be easily adapted to a given scenario, as we have previously experimented in [24]. We have chosen a fuzzy-based location estimator in order to determine the location of the dog according to the RSSI values measured by the Arduino device. The estimator is based on measuring the intensity of the received signal, something that can be done continuously, on a regular basis or on demand. Although many indoorg RF-based user location and tracking system, as RADAR [25], have been widely considered, we consider more appropriated for the scenario here described the use of fuzzy logic. Fuzzy logic is used to handle the uncertainties that might be encountered and provide systems that do not require a specialized hardware or a great number of sensors or devices. As RSSI values can easily vary due to many reasons, for example due to the influence of the own body, we need to deal with inexact and uncertain information, so fuzzy methods [24,26,27] are suitable for the scenario here described. The device measures the RSSI obtained from each ZigBee node of the flat (see Figure 11) and transmits the values and the identifiers (IDs) of the nodes to the sink node, where the middleware located at the mini-PC stores the data collected into the database and estimates the location of the dog according to the signal strength map previously obtained. The comparison of the expected RSS values with those obtained allow to estimate the pet location. The radio propagation analysis presented in this paper allows obtaining the signal strength map in a simpler and less labor-intensive way than classical methods of signal measuring [25,28,29]. The main restrictions of this method are the requisites of creating maps based on floor plans of indoors, of choosing the effective location of the (ZigBee) nodes used as beacons inside the building, and choosing the effective positioning technology and algorithms. Signal variation recommends using fuzzy techniques, since they perform better than crisp ones. Furthermore, the knowledge of the precise location often does not contribute to a better behavior of the location based systems. Fuzzy locating [24] determines the similarity (or even distance) between the RSS values obtained by the dog and the a priori knowledge with the operational ambience (the signal strength map of the flat). Tracking motion over time with the aid of a fuzzy automaton allows to increase the accuracy of the location estimator.

The location module has two main components, a set of fuzzy rules and a set of fuzzy automata. Rules are evaluated with the data collected and then location estimation is provided. An adaptive neuro-fuzzy inference system (ANFIS) has been used to obtain the rules. We have divided the flat in areas following a 8 × 10 mesh, and using the Matlab tool (fuzzy and neural network toolboxes) and the signal strength map previously obtained we have obtained a set of 63 rules as:

*S1 is High AND S2 is VeryLow AND S3 is High THEN location = Entry*

The obtention of the rules is simple, but the main problem is that the mechanism is totally dependent on the scenario considered. The cost of evaluation of fuzzy rules is very low since only a set of IF-THEN-ELSE sentences must be computed. Fuzzy automata require a bit more complicated calculus, since fuzzy operators are more complex, but not too much. That allows real-time operation. The fuzzy automata take advantage of the movement restriction, since the dog do not cross walls during its movement along the rooms of the flat. Thus, we define the potential travel routes that can be followed by the dog and we obtain a set of fuzzy automata. Each of the regions of the mesh previously defined is characterized as a symbol (character), and then a travel route is a string of symbols, where each symbol is the region where it has traveled. Tracking is performed by measuring the simuilarity between the string α representing the the path followed by the pet and the routes defined by the automata (ω_i_). More information can be obtained in [30].

**Figure 16:**
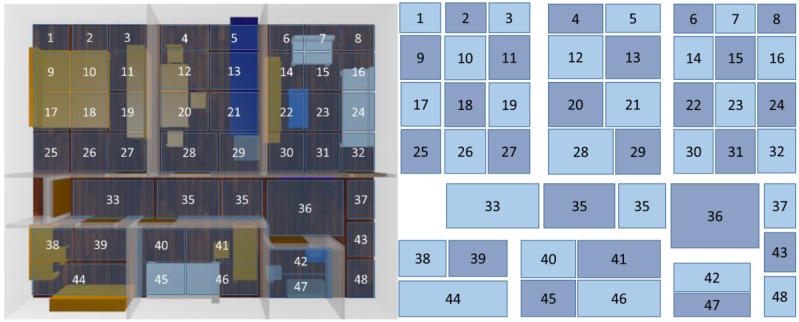
Grid distribution of the flat.

In order to validate the performance and accuracy of the system proposed by direct observation, we include a ground truth. We have fixed a forbidden area (see Figure 15a) and we have analyzed the behavior of the system during a day. Note the difficulty of the experimentation due to the limited cooperation of the dog. We have forced the dog to be placed in positions close to the forbidden room, but not entering it, either in front the door or the partitions. In the same way, we have forced the dog to enter the forbidden room and to sit and lie in different locations of this room. Results, depicted in Table 3, shows that the presence of the dog is incorrectly estimated 22.81% of the time, while it is succesfully determined the 77.19%. We analyze the presence (or not) of the dog in the forbidden area, determining the error/success rate of the estimations provided by the system. We define the success rate as the ratio between the number of times that the system has estimated that the dog was out of the room, when it was, and that the dog was in the room, when it was, and the total number of estimations (correct + incorrect estimations). In the same way, we define the error rate as the ratio between the number of incorrect estimations (the system has estimated that the dog was in the room, when it was out, and that the dog was out of the room, when it was in) and the total number of presence estimations. Incorrect estimations are due to both false positives (the dog has not entered the forbidden area, but the system so indicates) and false negatives (the dog has entered the forbidden area, but the system has not detected the intrusion), also addressed as type I and type II errors. The number of false positives and false negatives is quite similar, so any assumptions about them can be reached. As anticipated, the success rate obtained does not entirely satisfy the expected requirements, therefore we introduce the fuzzy automata. Fuzzy automata allow to discriminate impossible routes. Since the dog can only enter the room through the door (when opened) and the system permanently traces the location of the dog, the system corrects a great number of false positives and false negatives, resulting in a significantly higher success rate (close to 94.71%). Figure 16 depicts the distribution grid used by the fuzzy automata to track the movement of the dog and then discriminate type I and type II errors. The dog’s movement is modeled as a string of symbols, and the system builds a fuzzy automaton for each one of the possible sequences. Note that failing to pass through the walls significantly reduces the number of possible sequences. The string obtained is the concatenation of the grids traversed by the dog. Thus, the fuzzy automata compare the displacement followed by the dog with the set of all possible routes inside the flat and offers the value of similarity between them. The system selects the route that obtains a greater value of similarity allowing to correct many of the errors of type I and II.However, the error rate is not canceled (2.37% and 2.92%, respectively). The estimation errors concerning the door of the room are not discriminable. The automata are not able to determine whenever the door is opened or closed.

When estimating the time the pet spends at each location, it should be noted that the system periodically calculates the location of the pet, and this is what determines the estimation accuracy. The measurement period determines the granularity of the time estimation. As the sampling period is increased, the greater battery life of the ZigBee transceiver is achieved. Lengthening the sampling period involves a reduction on the accuracy of the estimation. If the dog moves between two consecutive observations, the system will not be aware of it. During the experimentation we have considered sampling periods of 5, 10, 30 and 60 s. Obviously, a sampling period of 5 s causes the transceiver battery runs out much earlier than in the case of considering a sample period of 60 s. A future work is to take advantage of the accelerometers located at motes to perform an intelligent choice of the sampling period. If the mote detects that our pet is not moving, it is not necessary to measure the RSSI level and this allows the ZigBee interface remains off longer, resulting in a lower battery consumption.

Pet monitoring, including the localization, tracking and time at forbidden areas of the pet (a dog in this case) has many similarities with the multiple existing proposals concerning human location tracking but the main difference occurs at the radio level. As it can be observed, the height and volume of the pet, which are significantly lower than those of people, motivates the RSSI values obtained. On one hand, the height plane in which lies the transceiver (in this case the transceiver is located in the dog’s neck), and on the other hand, the way it affects the volume of the pet to the received signal. The ZigBee routers (ZR) are located on furniture that does not exceed one meter high, so that ZigBee signals bounce off many obstacles and penetrate a variety of close materials that cause varying effects. This is evidenced by a decrease in the value of RSSI observed. People tend to find above mentioned obstacles, and in general, the effect of these elements in the signal propagation is less relevant than in the case of pets. The custom that many pets have of spending long periods of time huddled in a position without moving too much is another matter to be considered. When pets are snuggled, they tend to tilt the neck to its body, which, depending on the orientation of the pet’s transceiver to the ZR may cause observable changes in the RSSI values obtained. These variations, up to 5 dB, may be due to the directivity of the transceiver antenna, the ground effect, or even to the own body ot the pet. When the dog is snuggled, we observe average variations of RSSI values of 2.4 dBm and a maximum deviation of 5.1 dBm. Experimental results obtained do not allow to conclude that the animal’s movement affects (impede or favor) its location. Something that seems reasonable, because the dimensions of the apartment do not allow the pet to run and achieve a significant speed.

## 4. Discussion

In this work a novel indoor canine monitoring system based on WSN is presented. Thanks to this system, areas of the home that the dog has visited during the day can be easily known using any android based device, and furthermore, it can be monitored if the dog has entered into forbidden areas.

Since the system works considering the received power to carry out triangulation calculus, the deployment of the WSN network must be adequate and at least three routers must be visible for the device in any part of the home. In this work a 3D Ray Launching simulation technique is proposed for the proper deployment of the network and to calibrate application, since the power level in the different parts of the home for the three antennas must be registered in the application database.

Presented simulation tool can consider all parts of a complex scenario with the dielectric properties of all objects inside it and therefore, accurate results can be obtained if all parameters are correctly introduced. Besides, a canine simplified model compatible with the simulation code is developed for this work, which considering that the receiver is attached to the pet, is essential for the obtaining of accurate results. This model is flexible and allows the consideration of different kind of dogs and positions, taking under consideration all their properties.

BER results are also extracted from the simulation tool. This kind of results can help to find problems in the network as a consequence of interferences, since low error probability areas can be defined. These areas depends on the noise, transmission bit rate and the received power which depends on the transmitter antenna and the characteristics of the scenario. The radio planning analysis both in terms of path loss calculation as well as in terms of BER requirements show that for the type of scenario under analysis, mote deployment can be achieved with a node density of (1node/30–40 m^2^), for the selected XBee transceivers. Other communication systems could be employed, as a function of future requirements such as required transmission speed or delay constraints derived by transmission of non-delay tolerant traffic.

In any case, simulation results are compared in Figure 9 and Figure 10 with a campaign of measurements carried out inside the scenario, validating the simulation tool as well as the simplified canine model. Those results allow using 3D ray launching simulation to design and develop pet indoor location systems minimizing the campaign of measurements carried out inside the scenario considered. The software development cycle is significantly reduced with the use of simulation data for the extraction of the location rules, and the definition of the fuzzy automata. The power distributions obtained from the 3D ray Launching model allow configuring the location module without requiring the use of expensive fingerprinting techniques. Since one of the main problems of indoor RF location systems is the need to rebuild the system whenever the working scenario changes, the simulation tool allows to recalculate the location system on a quick, easy and simple way. The location and monitoring system has been implemented and tested in order to validate location capabilities as well as connectivity of the system (and hence, interaction capabilities) for the scenario under analysis.

## 5. Conclusions

In this work the viability of using a WSN network for dog monitoring is studied through a 3D Ray Launching simulation tool. In order to analyze the proposed system a simplified dog model has been developed considering different dog size proportions and dielectric properties of its biological tissues, in an automated way. A one-story home has been chosen as studied scenario considering its complete topology and characteristics. The main goal is the analysis of the influence of wireless propagation limitations in the implementation of a specific pet monitoring system, which has been implemented and tested.

A simplified parameterized dog model is implemented within the deterministic 3D RL code and introduced within the scenario under analysis in order to validate simulation results, proving to be an adequate approach in order to perform radioplanning tasks within the specific pet monitoring application. The estimations provide assessment in terms of required node density as well as on potential transceiver location, in order to comply with coverage/capacity requirements. Different measurement points are chosen and compared obtaining a maximum error of 5 dB in terms of RSSI detection error offset, demonstrating the accuracy of the 3D RL simulation method ane enabling node location tasks without previous measurement campaigns. In order to implement the pet monitoring system, an Android based indoor dog monitoring application has been coded in order to operate with XBee motes and a MeeGo mini PC, providing pet monitoring capabilities within the domestic household including a pet location algorithm based on fuzzy logic estimation. The proposed solution can be scaled in order to provide interactive communication with different pets and in different types of scenarios, with low deployment cost.

## Figures and Tables

**Table 1:** Animal monitoring system comparison.

System	Architecture	Communication System	Functionalities	Detected Variables	Reference
Cow activity monitoring system	Operator based	GSM, Bluetooth	Monitoring walking and milking activity	GPS, camera images	[8]
Animal Monitoring System (AHM)	Off-line	ZigBee	Monitoring physiological and environmental parameters	Humidity, Temperature, Heart Rate, Acceleration	[9]
Livestock Monitoring System (LMS)	Off-Line	Unknown	Monitoring all farm beasts transactions and products	GPS, Product tracking	[10]
Canine health monitoring system	Off-Line	Unknown cBAN (canine Body Area Network) system	Monitoring vital signs of dogs	ECG, PPG, GPS	[11]
Veterinary electrocardiographic monitoring system	Off-Line	Bluetooth	Monitoring vital signs in veterinary medicine	ECG	[12]
Solid dosing system for feeding dogs	Operator based	GSM	Feeding dogs with remote communication module	Food quantity	[13]
Pet Buddy	Off-Line	Bluetooth	Canine behavior recognition	Inertia	[14]
Cyber-Enhanced Working Dog (CEWD)	Off-Line	Wi-Fi	Integrated system for search and rescue	ECG, PPG, Gas, GPS, Inertia	[15]

**Table 2:** Simulation parameters used in the 3D Ray Launching code.

Parameter	Value
Frequency	2.41 GHz
Transmitted Power Level	0 dBm
Antenna gain	5 dBi
Vertical plane angle resolution Δθ	1°
Horizontal plane angle resolution Δφ	1°
Reflections	5

**Table 3:** Performance evaluation of the Location system.

*Location Evaluation*		Presence	Tracking
**Success**		**423**	**519**
**Error**		**125**	**29**
	*False positive (type I)*	*57*	*13*
	*False negative (type II)*	*68*	*16*

## Abbreviations

WSN	Wireless Sensor Networks
ISM	Industrial, Scientific and Medical
IoT	Internet of Things
PPG	Photophethysmogram
ECG	Electrocardiogram
GO	Geometrical Optics
GTD	Geometrical Theory of Diffraction
RFID	Radio Frequency IDentification
WPAN	Wireless Personal Area Networks
RSS	Received Signa Strength
RSSI	Received Signa Strength Indicator
PC	Personal Computer
RAM	Random Access Memory
USB	Universal Serial Bus
ANFIS	Adaptive Neuro-Fuzzy Inference System
